# mMWeb - An Online Platform for Employing Multiple Ecological Niche Modeling Algorithms

**DOI:** 10.1371/journal.pone.0043327

**Published:** 2012-08-17

**Authors:** Huijie Qiao, Congtian Lin, Liqiang Ji, Zhigang Jiang

**Affiliations:** 1 Key Laboratory of Animal Ecology and Conservation Biology, Institute of Zoology, Chinese Academy of Sciences, Beijing, China; 2 Graduate University of Chinese Academy of Sciences, Beijing, China; Institut Pluridisciplinaire Hubert Curien, France

## Abstract

**Background:**

Predicting the ecological niche and potential habitat distribution of a given organism is one of the central domains of ecological and biogeographical research. A wide variety of modeling techniques have been developed for this purpose. In order to implement these models, the users must prepare a specific runtime environment for each model, learn how to use multiple model platforms, and prepare data in a different format each time. Additionally, often model results are difficult to interpret, and a standardized method for comparing model results across platforms does not exist. We developed a free and open source online platform, the multi-models web-based (mMWeb) platform, to address each of these problems, providing a novel environment in which the user can implement and compare multiple ecological niche model (ENM) algorithms.

**Methodology:**

mMWeb combines 18 existing ENMs and their corresponding algorithms and provides a uniform procedure for modeling the potential habitat niche of a species via a common web browser. mMWeb uses Java Native Interface (JNI), Java R Interface to combine the different ENMs and executes multiple tasks in parallel on a super computer. The cross-platform, user-friendly interface of mMWeb simplifies the process of building ENMs, providing an accessible and efficient environment from which to explore and compare different model algorithms.

## Introduction

Ecological niche models (ENMs) [1]–[8] calculate the environmental conditions suitable to a given species using known occurrence data and environmental variables as an intermediate step to predicting its potential geographic distribution [6], [8]. Traditional and new theories, along with technical innovations are used in ecological niche modeling, and a wide variety of ENM platforms have been developed [4], [9]–[12]. Several toolkits are widely used for predicting species’ potential habitat [13]–[17]. Thus, ENM has become an important tool and is one of the frontiers for global change ecology, conservation biology, and biogeographical research during the last two decades [4].

Even though the internal methods of ENMs are different, ENMs have a similar modeling process that includes inputting species occurrence and environmental data, setting up a group of parameters, and model training and prediction of suitable habitat in a studied area [18]. However, each ENM has its own requirements in terms of data preparation (e.g. the format of input, environmental layers, and the parameters), system requirements (e.g. Java, MS Windows, MatLab script, and R environment) and requires users to have the necessary skills to implement the ENM [12]. The output values and file formats of the ENMs often differ, resulting in further confusion for the user. In addition, although each toolkit has its own method to evaluate the model accuracy (e.g. AUC/Kappa/TSS) [19], there is not a universal method to compare the results among models from different toolkits in a standardized environment.

Currently, some platforms exist that address some of these concerns. openModeller, which is an ecological niche modeling library, provides a uniform method for modeling potential habitat patterns using eleven modeling algorithms [18]. Lifemapper provides a simple interface to build the models in openModeller on a super computer [20]. Another computer platform, BIOMOD examines species-environment relationships under the R environment, providing eight algorithms [19]. While these platforms provide a variety of algorithm options to the user, little overlap exists between platforms. For example, openModeller and BIOMOD share only two algorithms, Artificial Neural Networks and Random Forests, whereas MaxEnt, which is widely used in this field, is not included in either platform at this time [21]. Therefore, the user must configure the openModeller, R environment for BIOMOD, and Java environment for MaxEnt if he (she) would like to execute multiple algorithms.

Furthermore, the output formats among openModeller, BIOMOD and MaxEnt differ. The openModeller uses the GeoTIFF format with output values between 0 to 255, while MaxEnt uses Arc/Info ASCII Grid format with float values between 0 to 1, and BIOMOD’s results are “data.frame" objects in R. Finally, the receiver operating characteristic (ROC) curve and the area under the curve (AUC) value, which is one of the most important evaluation indexes in ENMs [3], [22]–[26], is produced by different computational methods on each platform. The AUC value in BIOMOD is derived using a traditional method employing random pseudo-absent data [19], [27], while both openModeller and MaxEnt use an improved method, which replaces specificity with the proportion of area predicted present on the x-axis and thresholds sensitivity by subtracting a user defined omission error on the y-axis [3], [15], [21]. Although all the existing tools have AUC values corresponding to model results, it is not possible to compare model performance using the outputted AUC values directly.

In order to improve the efficiency of the ecological niche modeling process, we created mMWeb, a uniform platform to implement model runs and to compare model results derived from multiple algorithms. Construction of mMWeb began in 2008 during the implementation of the projects “Assessment on Threatened Status of Terrestrial Vertebrates in China" and “Monitoring the Biodiversity in Deserts and Grasslands." The objective of the mMWeb project is to provide web-based tools for researchers interested in using an ecological niche modeling approach to analyze species’ occurrence data, to display potentially suitable habitats across geographic space, and to compare multiple model algorithm results.

mMWeb provides a standard interface to invoke several existing applications (Table 1), to convert model results to the same format and scale, and to calculate and compare model accuracy between different algorithms using AUC values. mMWeb is a free, cross-platform, and open source project. Users can access the mMWeb platform via commonly used browsers, such as MS Internet Explorer, Firefox, Safari and Chrome. Additionally, all mMWeb application codes can be retrieved through a subversion repository.

**Table 1:** ENM platforms and associated algorithms available in mMWeb.

Full name	Abbreviation	Source	Literature(s)
Bioclimate Analysis and Prediction System	BioClim	openModeller	[12], [34]
Climate Space Model	CSMBS		
Ecological Niche Factor Analysis	ENFA		
Envelope Score	ENVSCORE		
Environmental Distance	ENVDIST		
Genetic Algorithm for Rule-set Production	GARP		
GARP Best Subsets	GARP_BS		
Support Vector Machine	SVM		
Artificial Neural Network	ANN	BIOMOD	[19], [35]
Classification Tree Analysis	CTA		
Generalized Additive Models	GAM		
Generalized Boosted Models	GBM		
Generalized Linear Models	GLM		
Multivariate Adaptive Regression Splines	MARS		
Random Forests	RF		
Maximum Entropy Modeling	MaxEnt	MaxEnt	[15]
Marble Algorithm	MA	mMWeb	[36]
Decision Tree Algorithm	DTA		

Eighteen existing algorithms (Table 1) are available in mMWeb. The mMWeb platform was built on existing open source software, including GDAL, GEOS, Proj.4, Weka, JQuery and can be deployed on any *nix based operating systems. The user interface was written in JavaScript. The web service and the task-scheduling component were written in Java based on a MySQL database. The interface, which is used to invoke the algorithms, was written in C++ and Java. Data analysis is carried out using R [28] and Weka for statistical analysis [29], GDAL for raster data analysis, and OGR for vector data analysis [30]. The results are stored in a GeoServer [31] and are rendered to our website via Open Geospatial Consortium (OGC) standards Web Map Service (WMS) through OpenLayers [32].

## Materials and Methods

**Figure 1:**
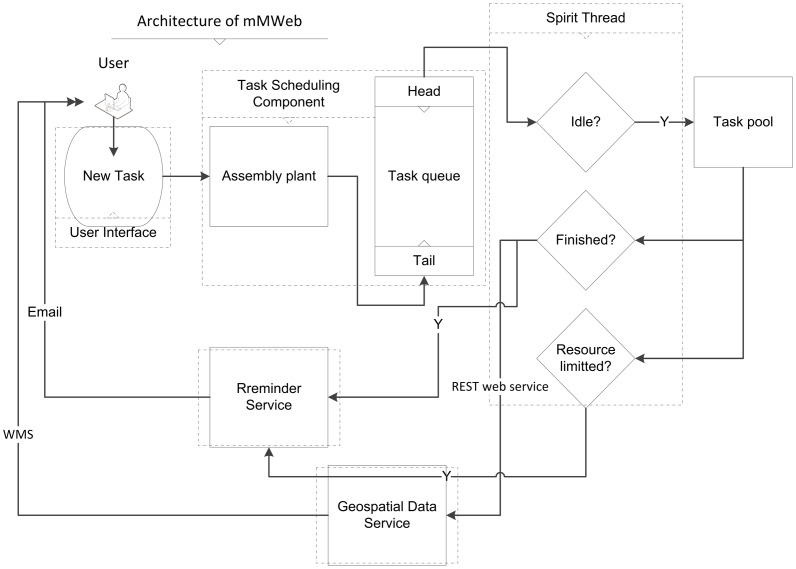
The flowchart of mMWeb. mMWeb consists of four independent modules: User interface, Task-scheduling component, Reminder service, and Geospatial data service.

mMWeb consists of four independent modules (Figure 1) and a User Center. The first module, the User Interface, creates a prediction task, monitors the task status, and displays results through a browser. The second module, the Task-scheduling Component, is the core of the platform. Within this module, the assembly plant assigns the occurrence records, selected models, and the associated parameters to a task, marks the task’s status as “in queue", and appends it to the tail of the task queue. The Spirit Thread is a memory-resident program. Whenever the task queue changes, the Spirit Thread is activated. If the number of the tasks in the task pool is less than the “MAXTASKS" parameter of mMWeb, the task at the head of the task queue will be moved to the task pool, and its status will be changed to “running". The tasks in the task pool are executed in parallel. When a task is completed, the Spirit Thread is activated to complete the final steps. These steps include converting model result formats, changing the task’s status to “finished", and clearing the temporary files and recovery resources.

Upon completion of a task, the Spirit Thread activates the third module, the Reminder Service, to send an email to the user. The Spirit Thread monitors the task continuously when it is in the task pool. If a task in the pool claims more resources than the limitations (i.e. maximum memory allocation or maximum execution time), the Spirit Thread will stop the execution of the task and inform the Reminder Service to send an email alert to the corresponding user.

The last module, the Geospatial Data Service, is an independent component. The module stores, analyzes, and displays task results. When the task is finished, the results are sent to the Geospatial Data Service via a Representational State Transfer (REST) interface. The user interface provides a function for exploring species occurrence records and model results via Web Map Service (WMS) in a browser.

**Figure 2:**
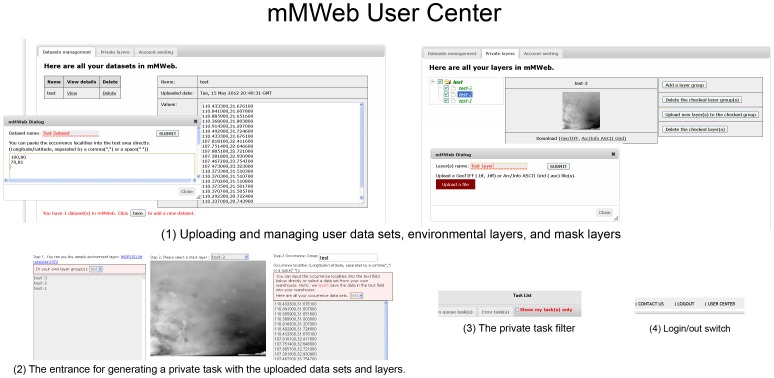
The User Center. The User Center includes the following functions: (1) Uploading and managing user data sets, environmental layers, and mask layers; (2) The entrance for generating a private task with the uploaded data sets and layers; (3) The private tasks filter; and (4) Login/out switch.

In addition to the geospatial data service, the User Center is also an independent component in mMWeb (Figure 2). It is a personal space that allows users to create their own accounts and to upload user defined environmental layers and occurrence localities. Uploaded data and tasks implemented through personal accounts are not available to other users and are managed by the account holder. The User Center provides the possibility for individuals to build a comprehensive model with specific environmental factors at various spatial resolutions in mMWeb. Additionally, the User Center has the capacity to supply account holders with a secure space to store data and results. mMWeb will not share or employ uploaded data for any commercial or non-commercial use without the permission of the user.

## Results

### Modeling Reeves’ Pheasant - a Case Study

We used ecological niche models in mMWeb to predict the potential distribution of suitable habitat of the endangered species, Reeves’ pheasant (*Syrmaticus reevesii*). Reeves’ pheasant is endemic in central and eastern China. The current geographic distribution of Reeves’ pheasant is quite small, with most of the existing wild populations inhabiting broad leafed forests. Occurrence data consisted of 49 localities, provided by Prof. Zhengwang Zhang of Beijing Normal University.

Using ENMs to investigate endangered species provides an opportunity to answer several questions regarding potential habitats under present and future environmental conditions. Here, we use Reeve’s pheasant as an example species to demonstrate the utility of mMWeb tools, but do not elaborate on the specific ecological niche of the species as it relates to model results.

The following steps outline the procedure used to predict the potential distribution of suitable habitat for Reeves’ pheasant within the mMWeb platform. Initially, we selected the 18 available models in mMWeb for implementation, using default parameter settings. The 19 WorldClim Bioclimatic layers [33] with a 10’ resolution served as the environmental dataset, and we chose a global extent as a mask layer.

**Figure 3:**
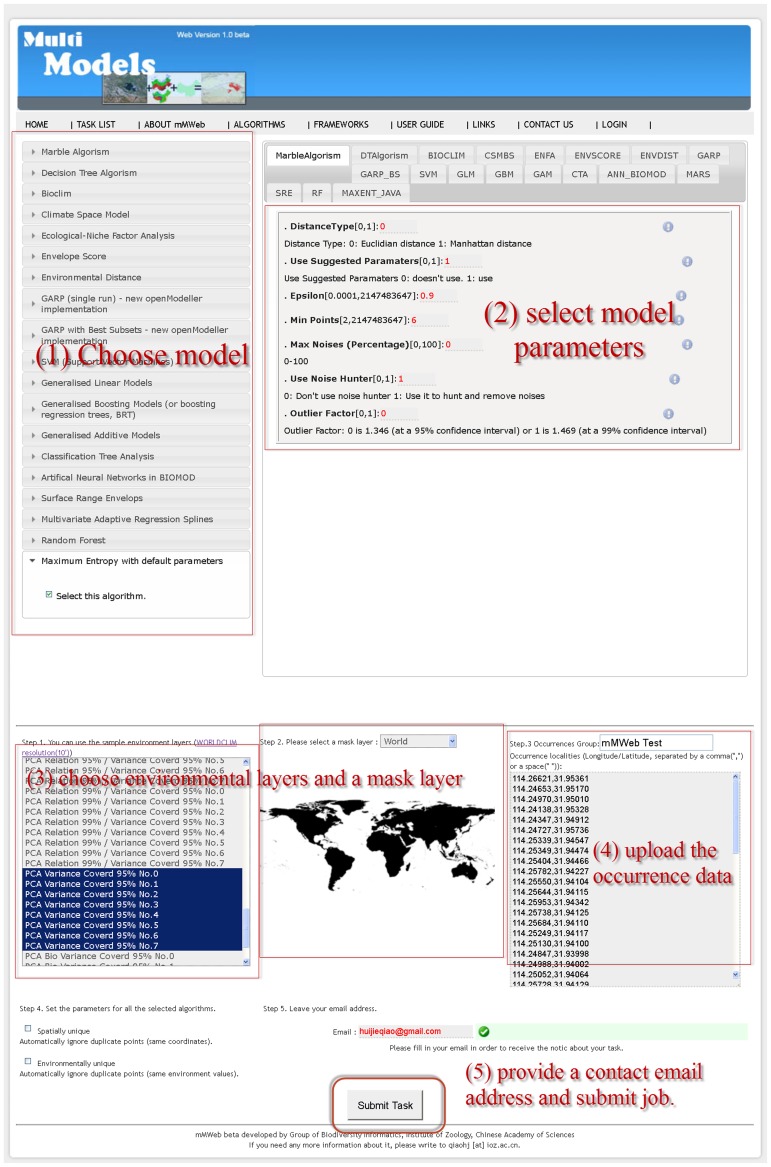
The steps to create and submit a task. 1) Choose model, 2) select model parameters, 3) choose environmental layers and a mask layer, 4) upload the occurrence data, 5) provide a contact email address and submit job.

Next we supplied an email address and clicked the “Submit job" button to invoke the tasks. We monitored the statuses of the tasks in the “Task List" page, although it is possible for the user to close the browser. When the tasks were completed, an email report was sent to the provided address with the option to download the archived results or to follow a web link that connected us to the results within mMWeb (Figure 3).

Upon completion of all tasks, the next objective was to compare results between models to determine the optimal algorithm under which to model the suitable habitat for Reeve’s Pheasant. Comparisons of ENMs are a hot topic in this field. However, there is not an acceptable general criterion for choosing a suitable algorithm for a data set. mMWeb standardizes all model results to values between 0 and 255 prior to calculating AUC values. This step provides the user with a means to compare model results across different algorithms. At present, mMWeb provides a widely used index, the receiver operator characteristic (ROC) and area under the curve (AUC) value, but also includes two improved methods for calculating AUC values [3], [22].

**Figure 4:**
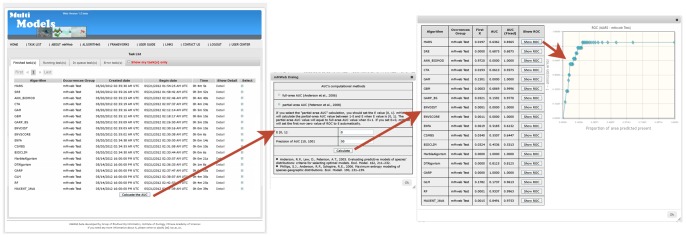
The steps to calculate the AUC value and show the ROC of task(s). 1) Select calculated results, 2) choose AUC method from menu, 3) select parameters, 4) calculate, and 5) show ROC.

In this case study, we selected all the finished tasks and calculated the partial-area AUC value with the parameters E = 0 and Precision of AUC = 50. Figure 4 shows the introduction of this function, the AUC values, and one ROC chart. In the case of Reeve’s pheasant, GBM, ENVSCORE, RF, GARP, MaxEnt, and MA had higher AUC values, and therefore, these algorithms would be appropriate to model the species.

**Figure 5:**
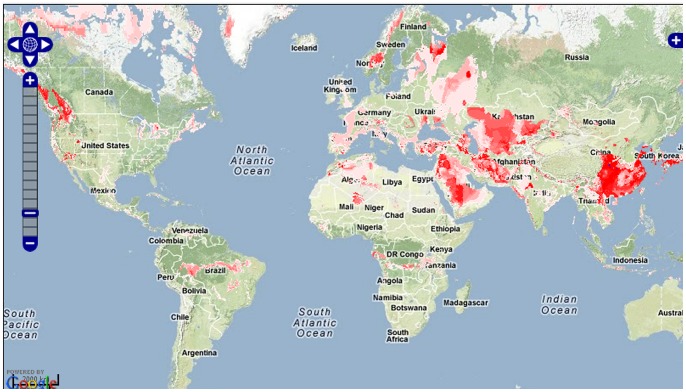
The Result of case study–Predicting the potential distribution of Reeves’ pheasant. The red dots represent the actual recorded Reeves’ pheasant distribution. The area in red represents the result of GARP in mMWeb. The habitat suitability increases as the red color changes from light red to red.

Figure 5 shows the GARP result after we clicked “Detail −> View report". The link “Download result" next to “View report" allows the user to download all the data, including the intermediate and final results of the corresponding model in a zip format file. A “readme.txt" in the downloaded zip file helps the user to repeat the modeling process in additional software platforms, such as MaxEnt, openModeller, WEKA and BIOMOD.

## Discussion

mMWeb provides solutions for those users interested in ecological niche modeling. It provides a user-friendly, web-based graphical environment for researchers and conservation managers. Users with little computer programming training can easily access and utilize several ENM algorithms available through mMWeb via a browser. All project tasks run continuously on a powerful server, and therefore, the modeling process is efficient.

mMWeb is an option for users who want to analyze large datasets. The system implements different algorithms and allows users to experiment with different modeling techniques without having to learn a new application, prepare data in a different format, or input data repeatably for each application.

The greatest contribution of mMWeb is the development of a platform from which the user can compare model results from different algorithms in a standardized format, thereby promoting the selection of an appropriate modeling approach for the specified dataset. Additionally, all task results can be directly transferred from mMWeb to other software, such as, ESRI ArcInfo, Weka, R, and MaxEnt.

### Availability and Future Directions

mMWeb is an open source platform (http://mmweb.animal.net.cn), freely available under the GNU Lesser General Public License. The PURL address of it is http://purl.oclc.org/mmweb. All source codes can be retrieved via a subversion repository (http://sourceforge.net/p/mmweb/code/2/tree/trunk/) or (svn://mmweb.animal.net.cn/mmweb/trunk) (Recommended).

Ecological niche modeling is a rapidly developing technique to address various questions in biology and paleontology. Therefore, we will continue to update the mMWeb platform with new algorithms and techniques to facilitate the modeling process. Additionally, the authors are working toward the inclusion of an algorithm selector, an innovative component with the ability to analyze a data set’s characteristics and to select the appropriate model(s) and associated parameter(s) based on these attributes.
